# Breast Cancer Screening Using a Modified Inertial Projective Algorithms for Split Feasibility Problems

**DOI:** 10.1155/2023/2060375

**Published:** 2023-09-07

**Authors:** Pennipat Nabheerong, Warissara Kiththiworaphongkich, Watcharaporn Cholamjiak

**Affiliations:** ^1^Radiology Department, School of Medicine, University of Phayao, Phayao 56000, Thailand; ^2^Radiology Department, Phayao Hospital, Phayao 56000, Thailand; ^3^School of Science, University of Phayao, Phayao 56000, Thailand

## Abstract

To detect breast cancer in mammography screening practice, we modify the inertial relaxed CQ algorithm with Mann's iteration for solving split feasibility problems in real Hilbert spaces to apply in an extreme learning machine as an optimizer. Weak convergence of the proposed algorithm is proved under certain mild conditions. Moreover, we present the advantage of our algorithm by comparing it with existing machine learning methods. The highest performance value of 85.03% accuracy, 82.56% precision, 87.65% recall, and 85.03% F1-score show that our algorithm performs better than the other machine learning models.

## 1. Introduction and Preliminaries

Breast cancer is common in women, with approximately 2 million diagnosed women worldwide each year [1]. Its mortality rate has increased over decades due to the change in risk factors, aging society, and better cancer registration and detection [2]. Many trials conclude that mammography screening at age 40s has mortality reduction, many years of life saved, and improved treatment, including evaluation of the extent of the disease [3]. However, there are some risks of this modality: overdiagnosis, false-positives, anxiety, and radiation injury [4]. Recently, imaging options combined with artificial intelligence are believed to be enhanced by integrating new screening protocols directed toward more personalized and precision medicine [1].

Many optimization algorithms were used to solve medical classification in machine learning; see [5, 6]. In this paper, we focus on the split feasibility problem (SFP) applying to mammography classification. Let *𝒞* and 𝒬 be two nonempty closed and convex subsets of real Hilbert space such that *𝒞*⊆*ℋ*_1_ and 𝒬⊆*ℋ*_2_, and let *𝒜* : *ℋ*_1_⟶*ℋ*_2_ be a bounded linear operator. The problem SFP is to

(1) $$\begin{array}{c} \text{find }\omega^{*}\in C\text{ such that }A\omega^{*}\in Q, \end{array}$$

if such *ω*^∗^ exist. The solution set *Ω*≔{*ω*^∗^ ∈ *𝒞* : *𝒜ω*^∗^ ∈ 𝒬} of the problem SFP (1) is denoted by *Ω*.

The first algorithm to solve the problem SFP (1) was presented by Censor and Elfving [7]. After that, many mathematicians (see [8–10]) applied the problem SFP (1) to solve many real-world problems such as machine learning, signal processing, image restoration, and many more. To find a solution of the problem SFP (1), Xu [11] proved that the point *ω*^∗^ is a solution of the problem SFP (1) if and only if the point *ω*^∗^ is a fixed point of the following mapping:

(2) $$\begin{array}{c} \text{Pro}{\text{j}}_{C}\left(I-\lambda \nabla f\right)=\text{Pro}{\text{j}}_{C}\left(I-\lambda A^{*}\left(I-Proj_{Q}\right)A\right). \end{array}$$

Later on, Byrne [12] was the first to propose a popular *𝒞*𝒬 algorithm for solving the problem SFP (1). The *𝒞*𝒬 algorithm of Byrne [12] was generated as follows:

(3) $$\begin{array}{c} \omega_{n+1}=\text{Pro}{\text{j}}_{C}\left(\omega_{n}-\lambda A^{*}\left(I-\text{Pro}{\text{j}}_{Q}\right)A\omega_{n}\right),\forall n\geq 1, \end{array}$$

where the parameter *λ* belongs in the interval (0, 2/‖*𝒜*‖^2^) which makes the mapping (*I* − *λ𝒜*^∗^(*I* − Proj_𝒬_)*𝒜* is nonexpansive where *I* is an identity, i.e.,

(4) $$\begin{array}{c} \left\|\left(I-\lambda A^{*}\left(I-\text{Pro}{\text{j}}_{Q}\right)Ax\right.-\left(I-\lambda A^{*}\left(I-\text{Pro}{\text{j}}_{Q}\right)Ay\right.\right\|\leq \left\|x-y\right\|,\forall x,y\in H_{1}, \end{array}$$

and *𝒜*^∗^ denotes for the adjoint operator of *𝒜*, while Proj_*𝒞*_ and Proj_𝒬_ are the orthogonal projections onto *𝒞* and 𝒬, respectively. The overall cost of calculation is not high if the metric projections onto *𝒞* and 𝒬 are simple to calculate. However, precisely computing the metric projection is difficult or requires too much effort in some circumstances when *𝒞* and 𝒬 are complex constructs.

Later on, Yang [13] introduced a relaxed *𝒞*𝒬 algorithm by modifying the *𝒞* and 𝒬 sets of the *𝒞*𝒬 algorithm (3) to reveal sets as follows:

(5) $$\begin{array}{c} C_{n}=\left\{x\in H_{1}:c\left(\omega_{n}\right)\leq \langle \xi_{n},\omega_{n}-x\rangle \right\}\;\text{and }Q_{n}=\left\{y\in H_{2}:q\left(A\omega_{n}\right)\leq \langle \eta_{n},A\omega_{n}-y\rangle \right\}, \end{array}$$

where *c* : *ℋ*_1_⟶ℝ ∪ {+∞} and *q* : *ℋ*_2_⟶ℝ ∪ {+∞} are two proper convex functions such that *ξ*_*n*_ ∈ *∂c*(*ω*_*n*_) and *η*_*n*_ ∈ *∂q*(*𝒜ω*_*n*_). Since the projections Proj_*𝒞*_*n*__ and Proj_𝒬_*n*__ are easier to use, many mathematicians use them to modify numerous algorithms for solving the problem SFP (1); see [14–16].

One of the techniques to speed up the convergence of the algorithms is the inertial technique which Polyak first introduced [17] in 1964. Polyak's algorithm was called the heavy ball method, and it was improved by Nesterov [18]. Later on, it has been widely used to solve a wide variety of problems in the optimization field, as seen in [9, 19–22].

In 2017, Dang et al. [9] modified the inertial technique which was introduced by Alvarez and Attouch [23] with the *𝒞*𝒬 algorithm of the Byrne algorithm (3) for the problem SFP (1) in a real Hilbert space. This algorithm was defined as follows:

(6) $$\begin{array}{c} \rho_{n}=\omega_{n}+\sigma_{n}\left(\omega_{n}-\omega_{n-1}\right), \end{array}$$

(7) $$\begin{array}{c} \omega_{n+1}=\text{Pro}{\text{j}}_{C_{n}}\left(\rho_{n}-\lambda A^{*}\left(I-\text{Pro}{\text{j}}_{Q_{n}}\right)A\left(\rho_{n}\right)\right),\forall n\geq 1, \end{array}$$

where the parameter *λ* is in the interval involving the norm of operator *𝒜*, *𝒞*_*n*_ and 𝒬_*n*_ are the revel set introduced by Yang [13], and the extrapolation factor $\sigma_{n}\in \left[0,{\bar{\sigma }}_{n}\right]$ and *σ* ∈ [0, 1) such that

(8) $$\begin{array}{c} {\bar{\sigma }}_{n}=\min\left\{\sigma ,\frac{1}{\max\left\{n^{2}{\left\|\omega_{n}-\omega_{n-1}\right\|}^{2},n^{2}\left\|\omega_{n}-\omega_{n-1}\right\|\right\}}\right\},\forall n\geq 1. \end{array}$$

The weak convergence of algorithm {*ω*_*n*_} generated by (6) was proved under the conditions of the extrapolation factor (8) and the stepsize parameter *λ*.

Very recently, Wang and Yu [32] generalized an inertial relaxed *𝒞*𝒬 of Yang [13] by modifying *𝒞*_*n*_, 𝒬_*n*_ as follows:

(9) $$\begin{array}{c} C_{n}=\left\{x\in H_{1}:c\left(\omega_{n}\right)\leq \langle \xi_{n},\omega_{n}-x\rangle -\frac{\alpha }{2}{\left\|\omega_{n}-x\right\|}^{2}\right\},\\ Q_{n}=\left\{y\in H_{2}:q\left(A\omega_{n}\right)\leq \langle \eta_{n},A\omega_{n}-y\rangle -\frac{\beta }{2}{\left\|A\omega_{n}-y\right\|}^{2}\right\}. \end{array}$$

*𝒞*_*n*_ and 𝒬_*n*_ of Wang and Yu [24] can be reduced to *𝒞*_*n*_ and 𝒬_*n*_ of Yang [13] when *α* and *β* are set to 0. The inertial generalized relaxed *𝒞*𝒬 algorithm (IGRCQ) of Wang and Yu [24] was introduced by *ω*_0_, *ω*_1_ ∈ *ℋ*_1_, and

(10) $$\begin{array}{c} \rho_{n}=\omega_{n}+\sigma_{n}\left(\omega_{n}-\omega_{n-1}\right), \end{array}$$

(11) $$\begin{array}{c} \omega_{n+1}=Proj_{C_{n}}\left(\rho_{n}-\lambda_{n}\nabla f_{n}\left(\rho_{n}\right)\right),\forall n\geq 1, \end{array}$$

where {*σ*_*n*_} ⊂ [0, *σ*) ⊂ [0, 1), {*ε*_*n*_} ⊂ (0, 4) and

(12) $$\begin{array}{c} \lambda_{n}=\left\{\begin{array}{cc} \varepsilon_{n}\frac{f_{n}\left(\omega_{n}\right)}{{\left\|\nabla f_{n}\left(\omega_{n}\right)\right\|}^{2}}, & \text{if }\left\|\nabla f_{n}\left(\omega_{n}\right)\right\|\neq 0,\\ 0, & \text{otherwise}. \end{array}\right. \end{array}$$

For each *n* ∈ *ℕ*, the functions are defined as follows:

(13) $$\begin{array}{c} f_{n}\left(\cdot \right)=\frac{1}{2}{\left\|\left(I-\text{Pro}{\text{j}}_{Q_{n}}\right)A\left(\cdot \right)\right\|}^{2}, \end{array}$$

(14) $$\begin{array}{c} \nabla f_{n}=A^{*}\left(I-\text{Pro}{\text{j}}_{Q_{n}}\right)A. \end{array}$$

It was shown that, under the conditions ∑_*n*=1_^∞^*σ*_*n*_‖*ω*_*n*_ − *ω*_*n*−1_‖^2^ < ∞ and liminf_*n*⟶∞_*ε*_*n*_(4 − *ε*_*n*_) > 0, the sequence {*ω*_*n*_} created by (10) weakly converges to a solution of the problem SFP (1).

In this paper, we modify the inertial technique with relaxed *𝒞*𝒬 algorithms and Mann's algorithm to solve the split feasibility problems in Hilbert spaces. We establish weak convergence theorems under suitable conditions. We apply our main result to solve a data classification problem by using extreme learning machine with mammographic mass data set from UCI, and then compare the performance of our algorithm with other existing machine learning algorithms.

## 2. Main Results

In this section, we introduce a new modified inertial projective algorithm by combining Mann algorithms with inertial technique and inertial relaxed *𝒞*𝒬 algorithm. Assume that *𝒞* and 𝒬 are two nonempty closed and convex subsets of real Hilbert space such that *𝒞*⊆*ℋ*_1_ and 𝒬⊆*ℋ*_2_ such that

(15) $$\begin{array}{c} C=\left\{\omega \in H_{1}:c\left(\omega \right)\leq 0\right\},Q=\left\{\omega \in H_{2}:q\left(A\omega \right)\leq 0\right\}, \end{array}$$

where *𝒜* : *ℋ*_1_⟶*ℋ*_2_ is a bounded linear operator, *c* : *ℋ*_1_⟶ℝ and *q* : *ℋ*_2_⟶ℝ are lower semicontinuous convex functions. We next assume that *∂c* and *∂q* are bounded operators. For a sequence {*y*_*n*_} in *ℋ*_1_, we modify the half-spaces *𝒞*_*n*_ and 𝒬_*n*_ by using the idea of Wang and Yu [24] as follows:

(16) $$\begin{array}{c} C_{n}=\left\{\omega \in H_{1}:c\left(y_{n}\right)\leq \langle \varrho_{n},y_{n}-\omega \rangle -\frac{\alpha }{2}{\left\|y_{n}-\omega \right\|}^{2}\right\}, \end{array}$$

where *ϱ*_*n*_ ∈ *∂c*(*y*_*n*_), *α* ≥ 0, and

(17) $$\begin{array}{c} Q_{n}=\left\{\omega \in H_{2}:q\left(Ay_{n}\right)\leq \langle \xi_{n},Ay_{n}-\omega \rangle -\frac{\beta }{2}{\left\|Ay_{n}-\omega \right\|}^{2}\right\}, \end{array}$$

where *ξ*_*n*_ ∈ *∂q*(*𝒜y*_*n*_) and *β* ≥ 0. We see that *𝒞*⊆*𝒞*_*n*_ and 𝒬⊆𝒬_*n*_ for each *n* ≥ 1. Define *f*_*n*_(·) and ∇*f*_*n*_(·) as in (13).

We now introduce a modified inertial projective algorithms as follows:

Assume that the following condition hold:

(C1) ∑_*n*=1_^∞^*σ*_*n*_max{‖*ω*_*n*_ − *ω*_*n*−1_‖^2^, ‖*ω*_*n*_ − *ω*_*n*−1_‖} < ∞; 

(C2) 0 < liminf_*n*⟶∞_*λ*_*n*_ ≤ limsup_*n*⟶∞_*λ*_*n*_ < 2/‖*𝒜*‖^2^;

(C3) 0 < liminf_*n*⟶∞_*α*_*n*_ ≤ limsup_*n*⟶∞_*α*_*n*_ < 1.

**Theorem 1 .**

Let *ℋ*_1_ and *ℋ*_2_ be two real Hilbert spaces, and let *𝒞* and 𝒬 be nonempty closed convex subsets such that *𝒞*⊆*ℋ*_1_ and 𝒬⊆*ℋ*_2_. Let *𝒜* : *ℋ*_1_⟶*ℋ*_2_ be a bounded linear operator. Assume that the solution set *Ω* of the problem SFP (1) is nonempty, the condition (C1)-(C2) hold. Then the sequence {*ω*_*n*_} generated by Algorithm 1:. converges weakly to a point *ω*^∗^ ∈ *Ω*.

**Proof**

Let *ω*^∗^ ∈ *Ω*. Since (*I* − *Proj*_𝒬_*n*__) is firmly nonexpansive, then for each *n* ∈ *ℕ*, we have

(18) $$\begin{array}{c} 2f_{n}\left(y_{n}\right)={\left\|\left(I-\text{Pro}{\text{j}}_{Q_{n}}\right)Ay_{n}\right\|}^{2}={\left\|\left(I-\text{Pro}{\text{j}}_{Q_{n}}\right)Ay_{n}-\left(I-\text{Pro}{\text{j}}_{Q_{n}}\right)A\omega^{*}\right\|}^{2}\leq \langle \left(I-\text{Pro}{\text{j}}_{Q_{n}}\right)Ay_{n}-\left(I-\text{Pro}{\text{j}}_{Q_{n}}\right)A\omega^{*},Ay_{n}-A\omega^{*}\rangle =\langle \left(I-\text{Pro}{\text{j}}_{Q_{n}}\right)Ay_{n},Ay_{n}-A\omega^{*}\rangle =\langle A^{*}\left(I-\text{Pro}{\text{j}}_{Q_{n}}\right)Ay_{n},y_{n}-\omega^{*}\rangle =\langle \nabla f_{n}\left(y_{n}\right),y_{n}-\omega^{*}\rangle . \end{array}$$

In the other hand, we set *t*_*n*_ = *y*_*n*_ − *λ*_*n*_∇*f*_*n*_(*y*_*n*_), From (18), we have

(19) $$\begin{array}{c} {\left\|\omega_{n+1}-\omega^{*}\right\|}^{2}={\left\|\left(1-\alpha_{n}\right)y_{n}+\alpha_{n}z_{n}-\omega^{*}\right\|}^{2}\leq \left(1-\alpha_{n}\right){\left\|y_{n}-\omega^{*}\right\|}^{2}+\alpha_{n}{\left\|z_{n}-\omega^{*}\right\|}^{2}\leq \left(1-\alpha_{n}\right){\left\|y_{n}-\omega^{*}\right\|}^{2}+\alpha_{n}\left({\left\|t_{n}-\omega^{*}\right\|}^{2}-{\left\|t_{n}-z_{n}\right\|}^{2}\right)={\left\|y_{n}-\omega^{*}\right\|}^{2}-\alpha_{n}{\left\|y_{n}-z_{n}\right\|}^{2}+2\alpha_{n}\lambda_{n}\langle \nabla f_{n}\left(y_{n}\right),y_{n}-z_{n}\rangle -4\alpha_{n}\lambda_{n}f_{n}\left(y_{n}\right)\leq {\left\|y_{n}-\omega^{*}\right\|}^{2}-\alpha_{n}{\left\|y_{n}-z_{n}\right\|}^{2}+2\alpha_{n}\lambda_{n}\left\|\nabla f_{n}\left(y_{n}\right)\right\|\left\|y_{n}-z_{n}\right\|-4\alpha_{n}\lambda_{n}f_{n}\left(y_{n}\right)\leq {\left\|y_{n}-\omega^{*}\right\|}^{2}+2\lambda_{n}^{2}\alpha_{n}{\left\|A\right\|}^{2}f_{n}\left(y_{n}\right)-4\alpha_{n}\lambda_{n}f_{n}\left(y_{n}\right)={\left\|y_{n}-\omega^{*}\right\|}^{2}-4\lambda_{n}\alpha_{n}\left(1-\frac{1}{2}\lambda_{n}{\left\|A\right\|}^{2}\right)f_{n}\left(y_{n}\right), \end{array}$$

(20) $$\begin{array}{c} {\left\|y_{n}-\omega^{*}\right\|}^{2}={\left\|\rho_{n}-\lambda_{n}\nabla f_{n}\left(\rho_{n}\right)-\omega^{*}\right\|}^{2}\leq {\left\|\rho_{n}-\omega^{*}\right\|}^{2}+\lambda_{n}^{2}{\left\|\nabla f_{n}\left(\rho_{n}\right)\right\|}^{2}-2\lambda_{n}\langle \rho_{n}-\omega^{*},\nabla f_{n}\left(\rho_{n}\right)-\nabla f_{n}\left(\omega^{*}\right)\rangle \leq {\left\|\rho_{n}-\omega^{*}\right\|}^{2}+\lambda_{n}^{2}{\left\|\nabla f_{n}\left(\rho_{n}\right)\right\|}^{2}-\frac{2\lambda_{n}}{{\left\|A\right\|}^{2}}{\left\|\nabla f_{n}\left(\rho_{n}\right)\right\|}^{2}\leq {\left\|\rho_{n}-\omega^{*}\right\|}^{2}-\left(\frac{2\lambda_{n}}{{\left\|A\right\|}^{2}}-\lambda_{n}^{2}\right){\left\|\nabla f_{n}\left(\rho_{n}\right)\right\|}^{2}\leq {\left\|\omega_{n}-\omega^{*}\right\|}^{2}+2\sigma_{n}\langle \omega_{n}-\omega_{n-1},y_{n}-\omega^{*}\rangle -\left(\frac{2\lambda_{n}}{{\left\|A\right\|}^{2}}-\lambda_{n}^{2}\right){\left\|\nabla f_{n}\left(\rho_{n}\right)\right\|}^{2}. \end{array}$$

Replacing (20) into (19), we have

(21) $$\begin{array}{c} {\left\|\omega_{n+1}-\omega^{*}\right\|}^{2}\leq {\left\|\omega_{n}-\omega^{*}\right\|}^{2}+2\sigma_{n}\langle \omega_{n}-\omega_{n-1},y_{n}-\omega^{*}\rangle -\left(\frac{2\lambda_{n}}{{\left\|A\right\|}^{2}}-\lambda_{n}^{2}\right){\left\|\nabla f_{n}\left(\rho_{n}\right)\right\|}^{2}-4\lambda_{n}\alpha_{n}\left(1-\frac{1}{2}\lambda_{n}{\left\|A\right\|}^{2}\right)f_{n}\left(y_{n}\right). \end{array}$$

This implies that

(22) $$\begin{array}{c} \left(\frac{2\lambda_{n}}{{\left\|A\right\|}^{2}}-\lambda_{n}^{2}\right){\left\|\nabla f_{n}\left(\rho_{n}\right)\right\|}^{2}+4\lambda_{n}\alpha_{n}\left(1-\frac{1}{2}\lambda_{n}{\left\|A\right\|}^{2}\right)f_{n}\left(y_{n}\right)\leq {\left\|\omega_{n}-\omega^{*}\right\|}^{2}-{\left\|\omega_{n+1}-\omega^{*}\right\|}^{2}+2\sigma_{n}\langle \omega_{n}-\omega_{n-1},y_{n}-\omega^{*}\rangle . \end{array}$$

Since *I* − *λ*_*n*_∇*f*_*n*_ and Proj_*𝒞*_*n*__ are nonexpansive, we have

(23) $$\begin{array}{c} \left\|\omega_{n+1}-\omega^{*}\right\|=\left\|\left(1-\alpha_{n}\right)y_{n}+\alpha_{n}z_{n}-\omega^{*}\right\|\leq \left(1-\alpha_{n}\right)\left\|y_{n}-\omega^{*}\right\|+\alpha_{n}\left\|z_{n}-\omega^{*}\right\|\leq \left\|\rho_{n}-\omega^{*}\right\|\leq \left\|\omega_{n}-\omega^{*}\right\|+\sigma_{n}\left\|\omega_{n}-\omega_{n-1}\right\|. \end{array}$$

By Lemma 1 in [25] and (C1), we have that {‖*ω*_*n*_ − *ω*^∗^‖} is convergence sequence for any *ω*^∗^ ∈ *Ω*. Therefore, {*ω*_*n*_} is bounded. From the definition of {*ρ*_*n*_}, {*ρ*_*n*_} is also bounded. It follows from (20), (C1)-(C3) that

(24) $$\begin{array}{c} \underset{n⟶\infty }{\lim}\left\|\nabla f_{n}\left(\rho_{n}\right)\right\|=\underset{n⟶\infty }{\lim}f_{n}\left(y_{n}\right)=0. \end{array}$$

Again by *I* − *λ*_*n*_∇*f*_*n*_ and *P*_*𝒞*_*n*__ are nonexpansive, then we have

(25) $$\begin{array}{c} {\left\|\omega_{n+1}-\omega^{*}\right\|}^{2}={\left\|\left(1-\alpha_{n}\right)y_{n}+\alpha_{n}z_{n}-\omega^{*}\right\|}^{2}\leq {\left\|\rho_{n}-\omega^{*}\right\|}^{2}-\left(1-\alpha_{n}\right)\alpha_{n}{\left\|y_{n}-z_{n}\right\|}^{2}\leq {\left\|\omega_{n}-\omega^{*}\right\|}^{2}+2\sigma_{n}\langle \omega_{n}-\omega_{n-1},\rho_{n}-\omega^{*}\rangle -\left(1-\alpha_{n}\right)\alpha_{n}{\left\|y_{n}-z_{n}\right\|}^{2}, \end{array}$$

which implies that

(26) $$\begin{array}{c} \left(1-\alpha_{n}\right)\alpha_{n}{\left\|y_{n}-z_{n}\right\|}^{2}+2\sigma_{n}\langle \omega_{n-1}-\omega_{n},\rho_{n}-\omega^{*}\rangle \leq {\left\|\omega_{n}-\omega^{*}\right\|}^{2}-{\left\|\omega_{n+1}-\omega^{*}\right\|}^{2}. \end{array}$$

From lim_*n*⟶∞_‖*ω*_*n*_ − *ω*^∗^‖ exists, (C1) and (C3), we obtain

(27) $$\begin{array}{c} \underset{n⟶\infty }{\lim}\left\|y_{n}-z_{n}\right\|=0. \end{array}$$

It follows from (C2) and (24) that

(28) $$\begin{array}{c} \underset{n⟶\infty }{\lim}\left\|y_{n}-\rho_{n}\right\|=\underset{n⟶\infty }{\lim}\lambda_{n}\left\|\nabla f_{n}\left(\rho_{n}\right)\right\|=0. \end{array}$$

And it is clearly from (C1), we have

(29) $$\begin{array}{c} \underset{n⟶\infty }{\lim}\left\|\rho_{n}-\omega_{n}\right\|=\underset{n⟶\infty }{\lim}\sigma_{n}\left\|\omega_{n}-\omega_{n-1}\right\|=0. \end{array}$$

From (28) and (29), we obtain

(30) $$\begin{array}{c} \underset{n⟶\infty }{\lim}\left\|y_{n}-\omega_{n}\right\|=0. \end{array}$$

Finally, let *ω*^∗^ be a weak sequential cluster point of {*ω*_*n*_}. There exists a subsequence {*ω*_*n*_*k*__} of {*ω*_*n*_} which converges weakly to *ω*^∗^ ∈ *ℋ*_1_. From (30), we also have that {*y*_*n*_*k*__} converges weakly to *ω*^∗^ and hence *𝒜y*_*n*_*k*__⇀*𝒜ω*^∗^ as *k*⟶∞. By the definition of *z*_*n*_, we have that *P*_𝒬_*n*__(*Ay*_*n*_) ∈ 𝒬_*n*_. This implies that

(31) $$\begin{array}{c} q\left(Ay_{n_{k}}\right)\leq \langle \xi_{n_{k}},\left(I-\text{Pro}{\text{j}}_{Q_{n_{k}}}\right)Aa_{n_{k}}\rangle -\frac{\beta }{2}{\left\|\left(I-\text{Pro}{\text{j}}_{Q_{n_{k}}}\right)Aa_{n_{k}}\right\|}^{2}, \end{array}$$

where *ξ*_*n*_*k*__ ∈ *∂q*(*𝒜y*_*n*_*k*__). By our assumption as *∂q* is bounded (24) and (31), we have *q*(*𝒜ω*^∗^) ≤ 0, this shows that *𝒜ω*^∗^ ∈ 𝒬. Again, by the definition of {*z*_*n*_}, we have that *z*_*n*_*k*__ ∈ *𝒞*_*n*_*k*__. This implies that

(32) $$\begin{array}{c} c\left(y_{n_{k}}\right)\leq \langle \varrho_{n_{k}},y_{n_{k}}-z_{n_{k}}\rangle -\frac{\alpha }{2}{\left\|y_{n_{k}}-z_{n_{k}}\right\|}^{2} \end{array}$$

where *ϱ*_*n*_*k*__ ∈ *∂c*(*y*_*n*_*k*__). By our assumption as *∂c* is bounded (27) and (32), we have *c*(*ω*^∗^) ≤ 0, this shows that *ω*^∗^ ∈ *𝒞*. By Opial's lemma in [26], we can conclude that {*ω*_*n*_} converges weakly to a solution in *Ω*. This completes the proof.

## 3. Application to Data Classification Problem

Nowadays, many cancer patients are reported around the world each year. In the population survey in Global Cancer Statistics 2020, information was found that breast cancer was the most severe disease with 258 new cases per hour [27]. Breast cancer is more common in developed countries than in developing countries, and the number of cases varies with per capita income (GDP per capita). In addition, people's way of life in the city (urbanization) and those environments result in more risk behaviors for breast cancer. Importantly, it also found that the number of patients tends to increase significantly each year as well. In Thailand, breast cancer is the 1st most common cancer among females and the 3rd most common among both males and females. There are 8,266 deaths from breast cancer per year or about 1 person per hour. The above data shows that breast cancer is rapidly increasing and directly threatening the female population globally, including Thailand. In addition, the number of doctors specializing in such diseases is limited, not enough to provide services to the patients. Therefore, the use of technology-based knowledge related to artificial intelligence or machine learning including deep learning is the basis for creating tools or innovations that are efficient and accurate in assisting medical personnel in screening and diagnosing breast cancer. As a result, patients will be screened quickly and accurately, cured in the early stages and could reduce mortality. It is also an indicator of the modernization of the country's development in the field of public health in the future. Mammography plays a central part in the early detection of breast cancers because it can show changes in the breast years before a patient or physician can feel them. Research has shown that annual mammograms lead to early detection of breast cancers when they are most curable, and breast-conservation therapies are available. Women, beginning at age 40, should screen for mammography every year. In this research, we use the mammographic mass dataset from UCI is available on the UCI website. (https://archive.ics.uci.edu/ml/datasets/Mammographic+Mass?fbclid=IwAR1TL44iSKmqXX6PMiSVjqGVZRD-suQTPEVsejq01SUylZwildNu7UWEPZQ). This dataset contains a BI-RADS assessment, the patient's age, and three BI-RADS attributes: shape of mass, margin of mass, and density of mass together with the ground truth (the severity field) for 516 benign and 445 malignant masses that have been identified on full-field digital mammogram collected at the Institute of Radiology of the University Erlangen-Nuremberg between 2003 and 2006. These datasets can indicate how well a several computer-aided diagnosis (CAD) system performs compared to the radiologists [28]. After 167 missing attribute values from 7 BI-RADS, 5 ages, 31 shapes, 48 margins, and 76 densities were removed before the training process. The following Table 1 shows the overview of all attributes.

In 2021, Parvez et al. [29] showed many machine learning predictive models to classify breast cancer using this mammographic mass dataset. The following Table 2 shows the comparison of our algorithm 1 consider in two constrain closed convex sets *L*_1_ and *L*_2_ with machine learning predictive models by Parvez et al. [29] after feature engineering.

From Table 2, the results show that our algorithm 1 when constrain closed convex set by *L*_2_ was used gives the highest accuracy 85.03% after removing rows with missing values and outliers. We next explain how our algorithm 1 optimizes weight parameter in training data for machine learning. We focus on extreme learning machine (ELM) by using 5-fold cross-validation [30]. The ELM method is defined as follows: assume that *𝒰*≔{(*μ*_*s*_, *r*_*s*_): *μ*_*s*_ ∈ ℝ^*n*^, *r*_*s*_ ∈ ℝ^*m*^, *s* = 1, 2, ⋯, *N*} is a set of training data with *N* distinct samples such that *μ*_*s*_ is an input training data and *r*_*s*_ is a target. Finding optimal output weight using the output function is the objective of the ELM method. The following output function is for single-hidden layer feed-forward neural networks (SLFNs) with *M* hidden nodes:

(33) $$\begin{array}{c} O_{s}=\overset{M}{\underset{i=1}{\sum }}w_{i}V\left(\langle c_{i},\mu_{s}\rangle +e_{i}\right), \end{array}$$

where *𝒱* is an activation function and *c*_*i*_ and *e*_*i*_ are parameters of weight and finally the bias, respectively. The optimal output weight *w*_*i*_ at the *i*-th hidden node is found by setting the hidden layer output matrix *ℋ* as follows:

(34) $$\begin{array}{c} H=\left[\begin{array}{ccc} V\left(\langle c_{1},\mu_{1}\rangle +e_{1}\right) & \cdots & V\left(\langle c_{M},\mu_{1}\rangle +e_{M}\right)\\ \vdots & \ddots & \vdots \\ V\left(\langle c_{1},\mu_{N}\rangle +e_{1}\right) & \cdots & V\left(\langle c_{M},\mu_{N}\rangle +e_{M}\right) \end{array}\right]. \end{array}$$

We also assume an optimal output weight *w* = [*w*_1_^*T*^, ⋯,*w*_*M*_^*T*^]^*T*^ such that *ℋw* = *ℛ*, where *ℛ* = [*r*_1_^*T*^, ⋯,*r*_*N*_^*T*^]^*T*^ is the training target data. For solving linear system *ℋw* = *ℛ*, we use the least square problem when the *Moore-Penrose generalized inverse* of *ℋ* is not easy to find. To ovoid overfitting in the machine learning, we consider constrain least square problem in two different closed convex subsets of *ℋ* as follows:

(35) $$\begin{array}{c} \underset{\omega \in C_{1}}{\min}\left\{{\left\|H\omega -R\right\|}_{2}^{2}\right\},\\ \;\underset{\omega \in C_{2}}{\min}\left\{{\left\|H\omega -R\right\|}_{2}^{2}\right\}, \end{array}$$

where *𝒞*_1_ = {*x* ∈ *ℋ* : ‖*x*‖_1_ ≤ *γ*}, *𝒞*_2_ = {*x* ∈ *ℋ* : ‖*x*‖_2_^2^ ≤ *γ*} such that *γ* is regularization parameters. Setting *f*(*ω*) = 1/2‖(*I* − *Proj*_𝒬_)*ℋω*‖_2_^2^, *Q* = *Q*_1_ = *Q*_2_ = {*ℛ*}, *c*_1_(*ω*) = ‖*ω*‖_1_ − *γ*, *c*_2_(*ω*) = ‖*ω*‖_2_^2^ − *γ*, and *q*(*ω*) = 1/2‖*ω* − *ℛ*‖^2^ for our algorithm 1 to solve the problem (3.1).

We use four evaluation metrics: accuracy, precision, recall, and F1-score [31] as explained below for comparing the performance of the classification algorithms.

(36) $$\begin{array}{c} \text{Accuracy}=\frac{\text{TP}+\text{TN}}{\text{TP}+\text{FP}+\text{TN}+\text{FN}}\times 100\%.\\ \text{Precision}=\frac{\text{TP}}{\text{TP}+\text{FP}}\times 100\%.\\ \text{Recall}=\frac{\text{TP}}{\text{TN}+\text{FN}}\times 100\%.\\ \text{F}1-\text{score}=\frac{2\times \left(\text{Precision}\times \text{Recall}\right)}{\text{Precision}+\text{Recall}}, \end{array}$$

where TN is the true negative, FP is the false positive, FN is the false negative, and TP is the true positive.

For avoiding model overfitting, we consider accuracy and loss plots. This research, we use the following binary cross-entropy loss function:

(37) $$\begin{array}{c} \text{Loss}=-\frac{1}{m}\overset{m}{\underset{i=1}{\sum }}O_{i}\log{\hat{y}}_{i}+\left(1-O_{i}\right)\log\left(1-{\hat{O}}_{i}\right), \end{array}$$

where ${\hat{O}}_{i}$ is the *i*-th scalar value in the model output, *O*_*i*_ is the corresponding target value, and *m* is the number of scalar values in the model output.

For comparison with other existing methods from the literature, the necessary parameters of each algorithm are chosen in Table 3. The extrapolation parameter of algorithm 1 (*L*_1_, *L*_2_) and algorithm (10) is in the following from:

(38) $$\begin{array}{c} \sigma_{n}=\left\{\begin{array}{cc} \frac{\sigma }{n^{2}\max\left\{{\left\|\omega_{n}-\omega_{n-1}\right\|}^{2},\left\|\omega_{n}-\omega_{n-1}\right\|\right.}, & \text{if }n>N,\omega_{n}\neq \omega_{n-1},\\ \sigma & \text{otherwise}, \end{array}\right. \end{array}$$

where *N* is a number of iterations that we want to stop and $\sigma_{n}={\bar{\sigma }}_{n}$ for algorithm (6).

Sigmoid is set as an activation function with hidden nodes *M* = 160, and four evaluation metrics of each algorithm are shown in Table 4.

Table 4 shows that our algorithm 1 with constrain closed convex set *L*_2_ is the highest F1-score, precision, recall, and accuracy efficiency. Additionally, our algorithm 1 with constrain closed convex set *L*_1_ has the lowest number of iterations. The optimal-fitting of our algorithm 1 is shown by considering the training and validation loss with the accuracy.

**Figure 1:**
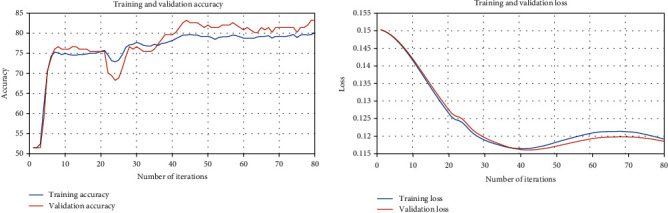
Training and validation loss and the accuracy plots of Algorithm 1: with constrain closed convex set *L*_1_.

**Figure 2:**
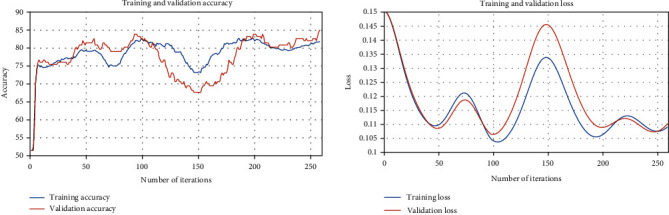
Training and validation loss and the accuracy plots of Algorithm 1: with constrain closed convex set *L*_2_.

From Figures 1 and 2, we observe that both of algorithm 1 *L*_1_ and *L*_2_ have optimal-fitting models. This means that the algorithm suitably learns the training dataset and generalizes well to predict the severity of mammographic mass based on BI-RADS assessment, the patient's age, shape, margin, and density of mass.

**Remark 1 .**

Since a matrix *ℋ* in ELM was generated by a finite dataset that contains real numbers, thus we can see from Table 4 that our Algorithm 1:. which requires a norm estimation of the bounded linear operator of *ℋ*, gives more efficiency than the algorithm (10) of Wang and Yu [24].

## 4. Conclusion and Discussion

Nowadays, there are many studies interested in the accuracy of artificial intelligence (AI) for the detection of breast cancer in mammography screening programme. Some believe that artificial intelligence (AI) has helped improve radiologists' performance and provides results equivalent or superior to those of radiologists' alone such as reduce the volume in screen-reading without affecting cancer detection substantially [32]. Although there are some issues that should be more explore including possible factors on recall and interval cancers [33], Freeman et al. [34] performed the systematic review of test accuracy and concluded that there is inadequate evidence in judgement of accuracy of artificial intelligence (AI) in detecting breast cancer on screening mammography. There is still small researches, which could not be representing the real effect of artificial intelligence (AI) in clinical practice or where on the clinical pathway AI might be of most benefit.

This paper presented an applying inertial modified relaxed *𝒞*𝒬 Mann algorithms for split feasibility problems for extreme learning machine based on BI-RADS assessment, the patient's age, and three BI-RADS attributes for predicting the severity of mammographic mass lesion to assist the physician regarding making decision about whether to go for biopsy or not. The comparison with other machine learning models and existing algorithms for split feasibility problems shows that our algorithm provides the highest performance value of 85.03% accuracy, 82.56% precision, 87.65% recall, and 85.03% F1-score. Moreover, considering training and validation loss, and the accuracy plots show that our algorithm has good fit model.

## Figures and Tables

**Algorithm 1:**
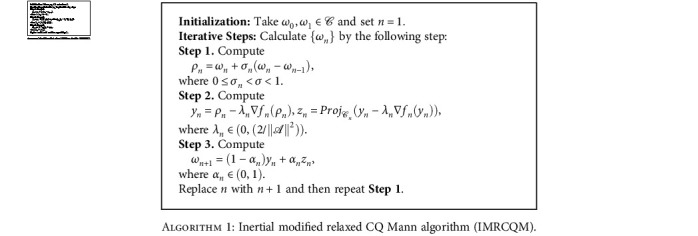
Inertial modified relaxed CQ Mann algorithm (IMRCQM).

**Table 1:** Overview of mammographic mass data set from UCI.

Attribute	Type	$\bar{x}$	Standard deviation	Max	Min	Coefficient of variation
BI-RADS	Double	4.33	0.63	6	1	14.65
Age	Double	55.78	14.67	96	18	26.30
Shape	Double	2.78	1.24	4	1	44.66
Margin	Double	2.81	1.57	5	1	55.71
Density	Double	2.92	0.35	4	1	12.04
Severity	Category					

**Table 2:** Highest accuracy of ML algorithms after feature engineering.

Machine learning model	Original dataset (%)	Highest accuracy (%)	Data cleaning or feature engineering
Logistic regression	80.46	82.50	Removing two least contributed features, RFE, or removing two least contributed features, correlation matrix
Linear discriminant analysis	78.90	84.50	Removing two least contributed features, RFE, or removing two least contributed features, correlation msatrix
K-nearest neighbors	79.03	82.05	Removing two least contributed features, RFE, or removing two least contributed features, correlation matrix
Classification and regression trees	74.48	82.97	Removing two least contributed features, RFE, or removing two least contributed features, correlation matrix
Gaussian Naive Bayes	78.37	83.73	Removing rows with missing values and outliers
Support vector machines	80.34	83.73	Removing two least contributed features, RFE, or removing two least contributed features, correlation matrix
Algorithm 1 (*L*_1_)	53.89	83.23	Removing rows with missing values and outliers
Algorithm 1 (*L*_2_)	53.89	**85.03**	Removing rows with missing values and outliers

**Table 3:** All different necessary parameters of each algorithm.

Parameter	*σ*	*λ*_*n*_	*α*_*n*_	*γ*	*λ*	*ε*_*n*_
Algorithm 1 (*L*_1_)	0.9999	$\frac{0.9999}{\left.\max\;\left.\text{eig}\left(A^{T}A\right)\right)\right)}$	$\frac{1}{1.2}$	7	—	—
Algorithm 1 (*L*_2_)	0.9999	0.9999/max eig(*A*^*T*^*A*)))	$\frac{1}{1.2}$	17	—	—
Algorithm (6) (*L*_1_)	0.9999	—	—	7	0.9999/max eig(*A*^*T*^*A*)))	—
Algorithm (6) (*L*_2_)	0.9999	—	—	17	0.9999/max eig(*A*^*T*^*A*)))	—
Algorithm (10) (*L*_1_)	0.9999	—	—	7	—	0.1
Algorithm (10) (*L*_2_)	0.9999	—	—	17	—	0.1

**Table 4:** All performances of each algorithm for comparison.

Algorithm	Number of iterations	Training time	Accuracy	Precision	Recall	F1-score
Algorithm (6) (*L*_1_)	410	0.1614	82.04	81.40	83.33	82.35
Algorithm (6) (*L*_2_)	271	0.1439	74.25	94.19	68.07	79.02
Algorithm (10) (*L*_1_)	188	0.2072	82.63	80.23	85.19	82.63
Algorithm (10) (*L*_2_)	242	0.3689	77.25	62.79	90.00	73.97
Algorithm 1 (*L*_1_)	**81**	0.0694	83.23	80.23	86.25	83.13
Algorithm 1 (*L*_2_)	259	0.2027	**85.03**	82.56	87.65	85.03

## Data Availability

The mammographic mass dataset from UCI is available on the UCI website (http://archive.ics.uci.edu/ml/datasets/mammographic+mass).
